# Blimp-1-Dependent IL-10 Production by Tr1 Cells Regulates TNF-Mediated Tissue Pathology

**DOI:** 10.1371/journal.ppat.1005398

**Published:** 2016-01-14

**Authors:** Marcela Montes de Oca, Rajiv Kumar, Fabian de Labastida Rivera, Fiona H Amante, Meru Sheel, Rebecca J. Faleiro, Patrick T. Bunn, Shannon E. Best, Lynette Beattie, Susanna S. Ng, Chelsea L. Edwards, Werner Muller, Erika Cretney, Stephen L. Nutt, Mark J. Smyth, Ashraful Haque, Geoffrey R. Hill, Shyam Sundar, Axel Kallies, Christian R. Engwerda

**Affiliations:** 1 QIMR Berghofer Medical Research Institute, Brisbane, Australia; 2 University of Queensland, School of Medicine, Brisbane, Australia; 3 Netaji Subhas Institute of Technology, New Delhi, India; 4 Queensland University of Technology, Institute of Health and Biomedical Innovation, Brisbane, Australia; 5 Griffith University, Institute of Glycomics, Gold Coast, Australia; 6 Griffith University, School of Natural Sciences, Nathan, Australia; 7 University of Manchester, Faculty of Life Sciences, Manchester, United Kingdom; 8 Walter and Eliza Hall Medical Research Institute, Division of Molecular Immunology, Melbourne, Australia; 9 The University of Melbourne, Department of Medical Biology, Melbourne, Australia; 10 Banaras Hindu University, Institute of Medical Sciences, Varanasi, Uttar Pradesh, India; University of Pennsylvania, UNITED STATES

## Abstract

Tumor necrosis factor (TNF) is critical for controlling many intracellular infections, but can also contribute to inflammation. It can promote the destruction of important cell populations and trigger dramatic tissue remodeling following establishment of chronic disease. Therefore, a better understanding of TNF regulation is needed to allow pathogen control without causing or exacerbating disease. IL-10 is an important regulatory cytokine with broad activities, including the suppression of inflammation. IL-10 is produced by different immune cells; however, its regulation and function appears to be cell-specific and context-dependent. Recently, IL-10 produced by Th1 (Tr1) cells was shown to protect host tissues from inflammation induced following infection. Here, we identify a novel pathway of TNF regulation by IL-10 from Tr1 cells during parasitic infection. We report elevated Blimp-1 mRNA levels in CD4^+^ T cells from visceral leishmaniasis (VL) patients, and demonstrate IL-12 was essential for Blimp-1 expression and Tr1 cell development in experimental VL. Critically, we show Blimp-1-dependent IL-10 production by Tr1 cells prevents tissue damage caused by IFNγ-dependent TNF production. Therefore, we identify Blimp-1-dependent IL-10 produced by Tr1 cells as a key regulator of TNF-mediated pathology and identify Tr1 cells as potential therapeutic tools to control inflammation.

## Author Summary

Many parasitic diseases are associated with the generation of potent inflammatory responses. These are often needed to control infection, but can also cause tissue damage if not appropriately regulated. IL-10 has emerged as an important immune regulator that protects tissues by dampening inflammation. Recently, some T cells that initially produce inflammatory cytokines have been found to start producing IL-10 as a mechanism of auto-regulation. We identified an important transcriptional regulator called B lymphocyte-induced maturation protein 1 (Blimp-1), which promotes IL-10 production by IFNγ-producing CD4^+^ T (Tr1) cells during malaria and visceral leishmaniasis, two important diseases caused by protozoan parasites. We found that Tr1 cell-derived IL-10 suppressed anti-parasitic immunity, but played a critical role in preventing tissue damage caused by the potent pro-inflammatory cytokine TNF. Specifically, IL-10 protected macrophages from TNF-mediated destruction, and this enabled lymphocytes to continue to migrate to regions in the spleen where T and B cell responses are generated. These findings allow us to better understand how parasites persist in a host, but also identify new opportunities to control inflammation to prevent disease.

## Introduction

TNF is a key pro-inflammatory cytokine required to control intracellular pathogens and kill tumours [1]. However, excessive TNF production can cause diseases such as rheumatoid arthritis, inflammatory bowel disease, psoriasis, ankylosing spondylitis, graft-versus-host disease and sepsis [2,3]. As such, TNF is a major target for the prevention of inflammatory diseases, and inhibitors of TNF activity are widely used in the clinic [3,4]. An important drawback to this approach is that it can increase susceptibility to infection, especially intracellular pathogens [5,6]. Therefore, a better understanding of how TNF is regulated during inflammation is needed to identify more selective ways to control disease while minimizing risk of infection.

CD4^+^ T cells play critical roles in coordinating immune responses by helping B cells produce high affinity antibodies, CD8^+^ T cells to kill infected and transformed cells and innate immune cells to recognize and control pathogens and tumour cells [7,8]. Many diseases caused by protozoan parasites require the generation of IFNγ- and TNF-producing CD4^+^ T (Th1) cells for the activation of macrophages and dendritic cells to kill captured or resident pathogens [9,10]. However, these potent pro-inflammatory cytokines, along with other T cell-derived cytokines such as IL-17, can also damage tissues, and as such, CD4^+^ T cell responses need to be tightly regulated so they themselves do not cause disease [11].

IL-10 is a major regulatory cytokine, and its secretion by conventional CD4^+^ T cells can suppress inflammation by directly inhibiting T cell functions, as well as upstream activities initiated by antigen presenting cells (APC’s) [12]. Initially, IL-10 production was identified in Th2 cells [13], but has since been described in Th1 [14–16], FoxP3-expressing regulatory T (Treg) [17,18] and IL-17-producing CD4^+^ T (Th17) [19] cell populations. Thus, CD4^+^ T cell-derived IL-10 production is emerging as an important mechanism to prevent immune pathology. In mice infected with protozoan parasites, Th1 cells are an important source of IL-10 that can promote parasite survival, but also limit pathology [20–28]. These IL-10-producing Th1 (Tr1) cells have also been identified in humans with visceral leishmaniasis (VL) caused by *Leishmania donovani* [29] and African children with *Plasmodium falciparum* malaria [30–32]. Tr1 cells are increasingly recognized as a critical regulatory CD4^+^ T cell subset that prevent immune pathology during disease and protect tissue from damage caused by excessive inflammation [12,33–35]. Despite these protective functions, Tr1 cells may also promote the establishment of infection [34] and suppress Th1-mediated, tumour-specific immunity [36]. However, it is not clear how much of this activity can be attributed to Tr1 cells or other IL-10-producing cell types. Therefore, a better understanding of IL-10 regulation by different cell types is required for the development of new therapeutic approaches targeting this cytokine.

Lymphoid tissue remodelling occurs in many chronic inflammatory settings associated with infectious, autoimmune and metabolic diseases [37–39]. This includes parasitic diseases such as malaria and VL that are associated with pronounced splenomegaly and disruption of lymphoid follicles [40,41]. In experimental models, this is accompanied by extensive vascular remodelling, white pulp atrophy and increased numbers of tissue macrophages [42–46], features also reported in human [47,48] and canine [49] VL. This remodelling results in dramatic changes to leukocyte movements in the spleen, and despite identifying excessive TNF production as a major contributor to these alterations [50,51], the immunoregulatory networks that fail are unknown.

Here, we identify a novel pathway of IL-10-dependent control of tissue pathology during parasitic infection. We show that IL-10 produced by Tr1 cells protects against IFNγ-dependent, TNF-mediated tissue damage, but limited the control of parasites that cause malaria and VL. This pathway is critically dependent on the transcriptional regulator B lymphocyte-induced maturation protein 1 (Blimp-1), which promotes IL-10 production by Tr1 cells. These findings provide new insights into the regulation and function of Tr1-derived IL-10, and thus reveal new opportunities to harness the therapeutic potential of these cells to protect against TNF-mediated diseases.

## Results

### Blimp-1 is required for CD4^+^ T cell IL-10 production in experimental malaria

**Fig 1:**
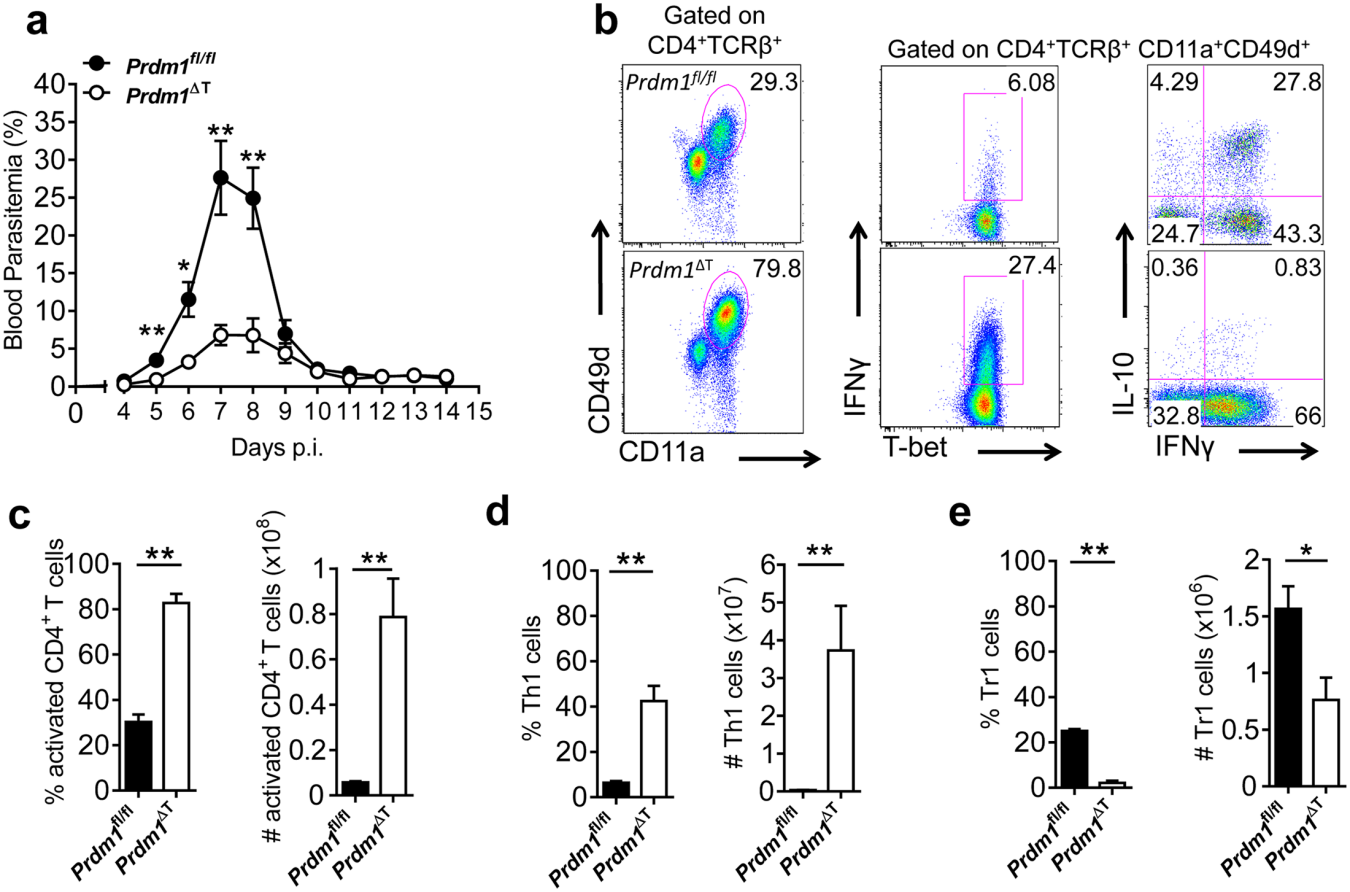
Blimp-1 inhibits the control of parasite growth during experimental malaria. **(A)** *Prdm1* ^fl/fl^ and *Prdm1* ^ΔT^ C57BL/6 mice were infected with PcAS and peripheral parasitemia was measured beginning at day 4 post infection (p.i.). (**B)** Splenic CD4^+^ T cell responses were assessed by flow cytometry, as indicated, at day 15 p.i.. The frequency and number of activated CD4^+^ T cells (CD11a^+^CD49d^+^) (**C)**, Th1 cells (IFNγ^+^Tbet^+^) (**D)** and Tr1 cells (IFNγ^+^IL-10^+^) (**E)** were measured. Representative of 3 independent experiments, mean ±SEM, n = 5 in each group in each experiment, **p<0.01, *p<0.05, Mann-Whitney U test.

The transcriptional regulator Blimp-1 (encoded by the *Prdm1* gene) has recently been implicated in the generation of IL-10-producing Tr1 cells [52,53]. To explore the relationship between Blimp-1 and IL-10 production in T cells during protozoan infections, we made use of *Prdm1* ^*fl/fl*^ x *Lck-Cre* C57BL/6 (*Prdm1* ^ΔT^) mice [54]. Strikingly, mice lacking Blimp-1 expression in T cells controlled non-lethal, *P*. *chabaudi* AS growth more efficiently than Cre-negative (*Prdm1* ^fl/fl^) litter mate controls (Fig 1A). This corresponded with an increased frequency and number of activated CD4^+^ T cells and Th1 cells, but severely impaired development of Tr1 cells in the spleen (Fig 1B–1E). A similar, but much smaller effect (50 fold less) was also seen in CD8^+^ T cells (S1 Fig). The pattern of immune response observed in control *Prdm1* ^fl/fl^ mice was similar to what we (S2 Fig) and others [23] observe in wild type C57BL/6 mice, suggesting the presence of the flox transgene is having minimal influence over immune responses. In addition, the changes described above in mice lacking Blimp-1 expression in T cells were observed as early as 4 days p.i., and clearly evident at day 7 p.i. (S3 Fig). When mice lacking Blimp-1 expression in T cells were infected with lethal *P*. *berghei* ANKA, they had reduced parasite burdens (S4A Fig), but despite delayed onset of severe disease and a small survival advantage, all mice ultimately succumbed with severe neurological symptoms (S4B Fig). Again, the reduced parasite burden in mice lacking Blimp-1 expression in T cells was associated with an increased frequency and number of Th1 cells, but impaired development of Tr1 cells (S4C and S4D Fig). Thus, Blimp-1 expression in T cells enhanced parasite growth and was critical for the generation of IL-10-producing Tr1 cells during experimental malaria.

### Elevated Blimp-1 expression and IL-10 production by CD4^+^ T cells is promoted by IL-12

**Fig 2:**
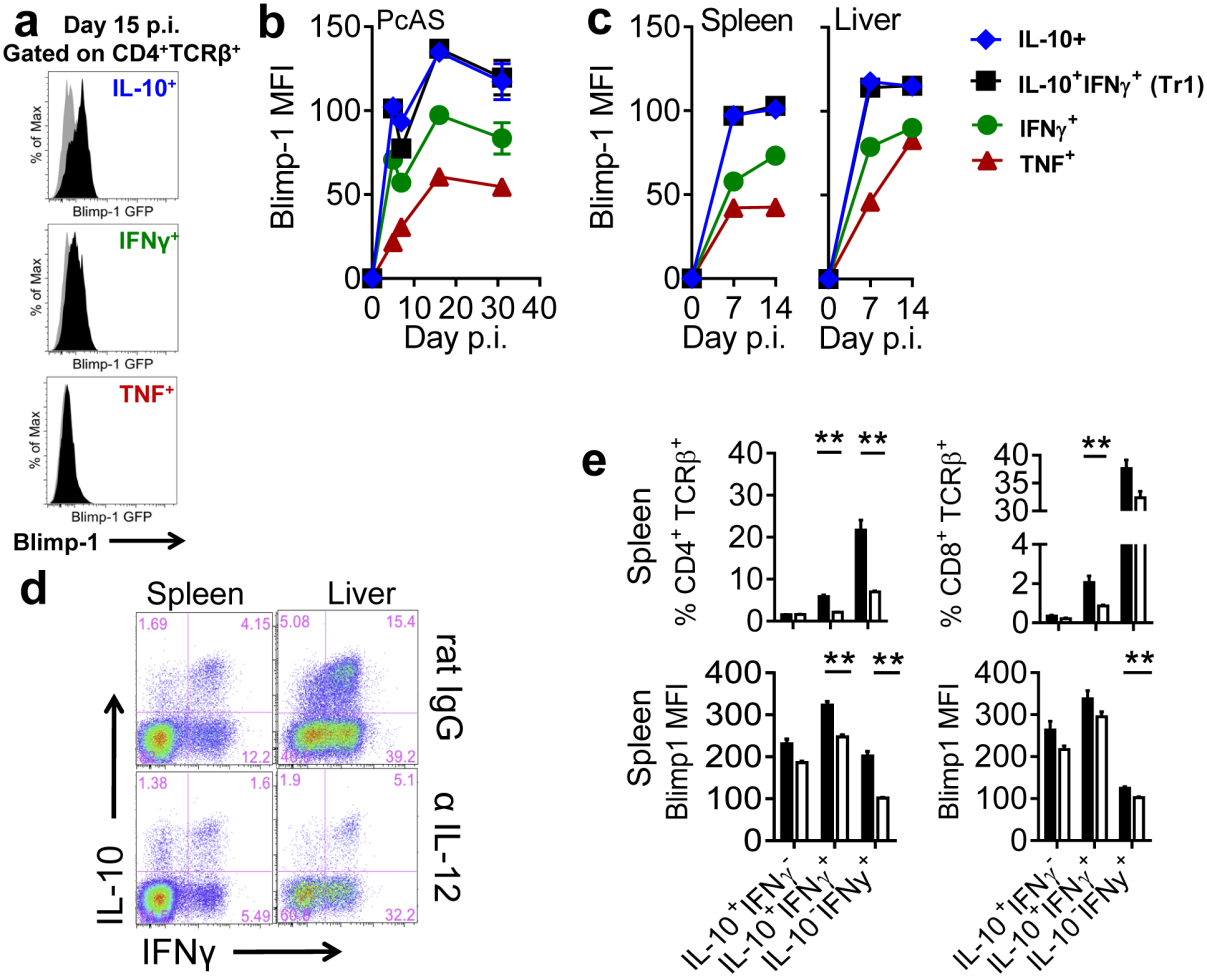
Blimp-1 controls CD4^+^ T cell derived IL-10 production. **(A)** *Prdm1* ^*GFP/+*^ (black-filled histograms) and WT (grey-filled histograms) mice were infected with PcAS. Blimp-1/GFP expression measured on CD4^+^ T cells producing IL-10, IFNγ and TNF at day 15 p.i.. (**B)** Kinetics of Blimp-1/GFP expression measured in various cytokine producing CD4^+^ T cells, as indicated, beginning at day 5 p.i.. **(C)** *Prdm1* ^*GFP/+*^ and WT mice were infected with *L*. *donovani* and the kinetics of Blimp-1 expression measured in the spleen and liver. (**D)** FACS plots showing gated CD4^+^ T cell IL-10 and IFNγ production from *Prdm1* ^*GFP/+*^ mice treated with 500 μg of polyclonal rat IgG (black bars in (**E**)) or anti-IL-12 (C17.8; white bars in (**E**)), as indicated, prior to infection and every 3 days following infection until day 14 p.i.. (**E**) The frequencies of IL-10 and IFNγ-producing CD4^+^ and CD8^+^ T cells, as well as Blimp-1 expressing CD4^+^ and CD8^+^ T cells, were determined by flow cytometry at day 14 p.i.. Representative of 3 similar experiments, mean ±SEM, n = 5 in each group in each experiment, **p<0.01, Mann-Whitney U test.

**Fig 3:**
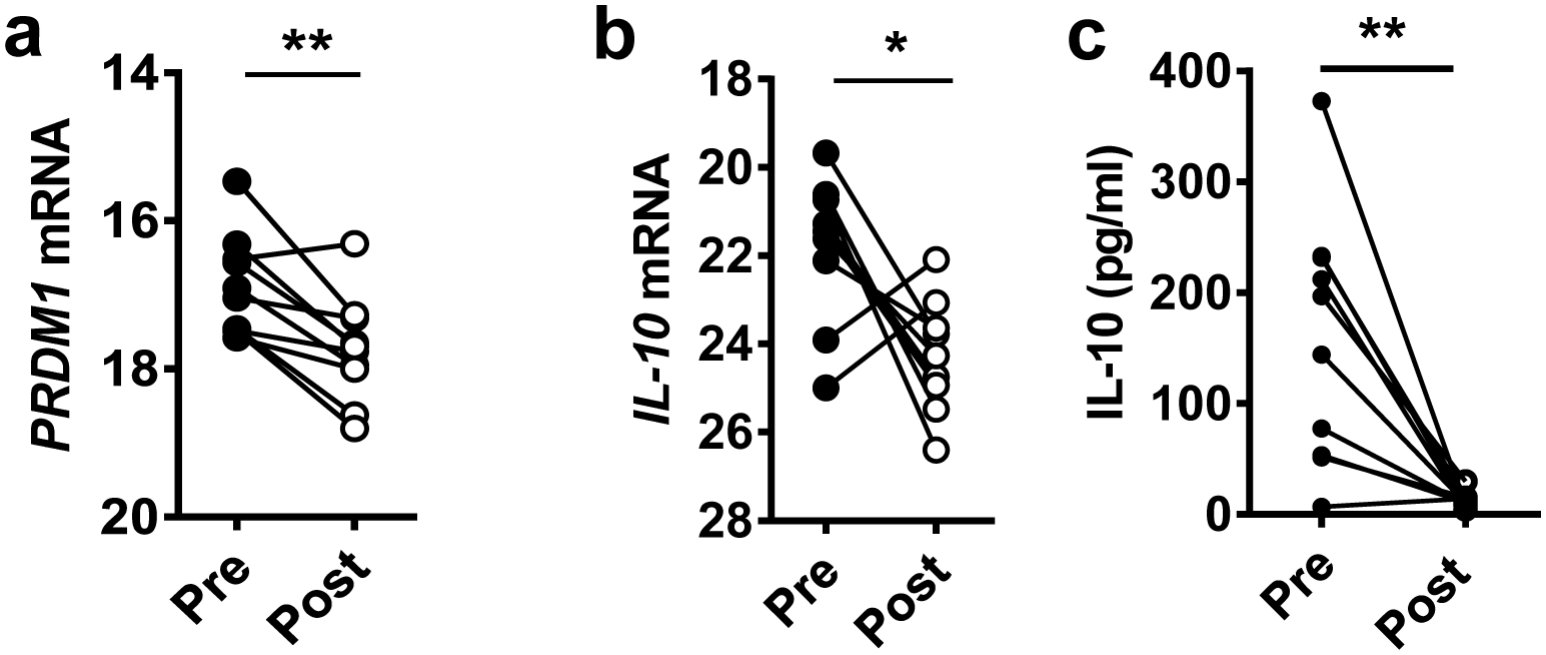
Elevated Blimp-1 levels in CD4^+^ T cells from VL patients were associated with increased IL-10 production. **(A)** *PRDM1* and (**B**) IL-10 mRNA accumulation in purified CD4^+^ cells isolated from the PBMCs, as well as (**C**) plasma IL-10 levels from VL patients before (closed circles; Pre) and 28 days after (open circles; Post) drug treatment. Data are from the same 10 paired samples, **p<0.01, *p<0.05, Wilcoxon signed-rank test.

We next examined Blimp-1 expression in CD4^+^ T cells using transgenic Blimp-1/GFP reporter mice [55] infected with *P*. *chabaudi* AS. Blimp-1 expression in CD4^+^ T cells was highest in IL-10-producing cells, lowest in TNF-producing cells, while those producing IFNγ expressed intermediate levels of Blimp-1 (Fig 2A and 2B). A similar pattern of association between CD4^+^ T cell cytokine production and Blimp-1 expression was found in mice with experimental VL caused by infection with the human protozoan parasite *L*. *donovani* (Fig 2C). Interestingly, there was no difference in Blimp-1 expression between CD4^+^ T cells expressing IL-10 alone and Tr1 cells in both infections (Fig 2B and 2C). Consistent with the finding that IL-12 is an important driver of Blimp-1-dependent Tr1 cell differentiation in autoimmunity [52], we found that Blimp-1 and IL-10 expression by IFNγ-producing CD4^+^ T cells from *L*. *donovani*-infected mice required IL-12 (Fig 2D and 2E). This reliance on IL-12 was most apparent for CD4^+^ T cells because although IL-12 blockade caused a small, but significant, reduction in IL-10 and IFNγ double-producing CD8^+^ T cell frequency, this was not accompanied by a significant reduction in Blimp-1 levels. In addition, we found no significant reduction in IFNγ-producing CD8^+^ T cells, although we did measure a small, but significant reduction in Blimp-1 levels (Fig 2E). Importantly, we found increased accumulation of *PRDM1* mRNA in CD4^+^ T cells isolated from the blood of VL patients, compared with the equivalent cell population in the same individuals after completion of drug treatment (Fig 3A). This was associated with elevated IL-10 mRNA levels in the same cells (Fig 3B), as well as increased plasma IL-10 (Fig 3C), as previously reported [29,56].

### Blimp-1-dependent IL-10 production by CD4^+^ T cells limits parasite killing in VL

**Fig 4:**
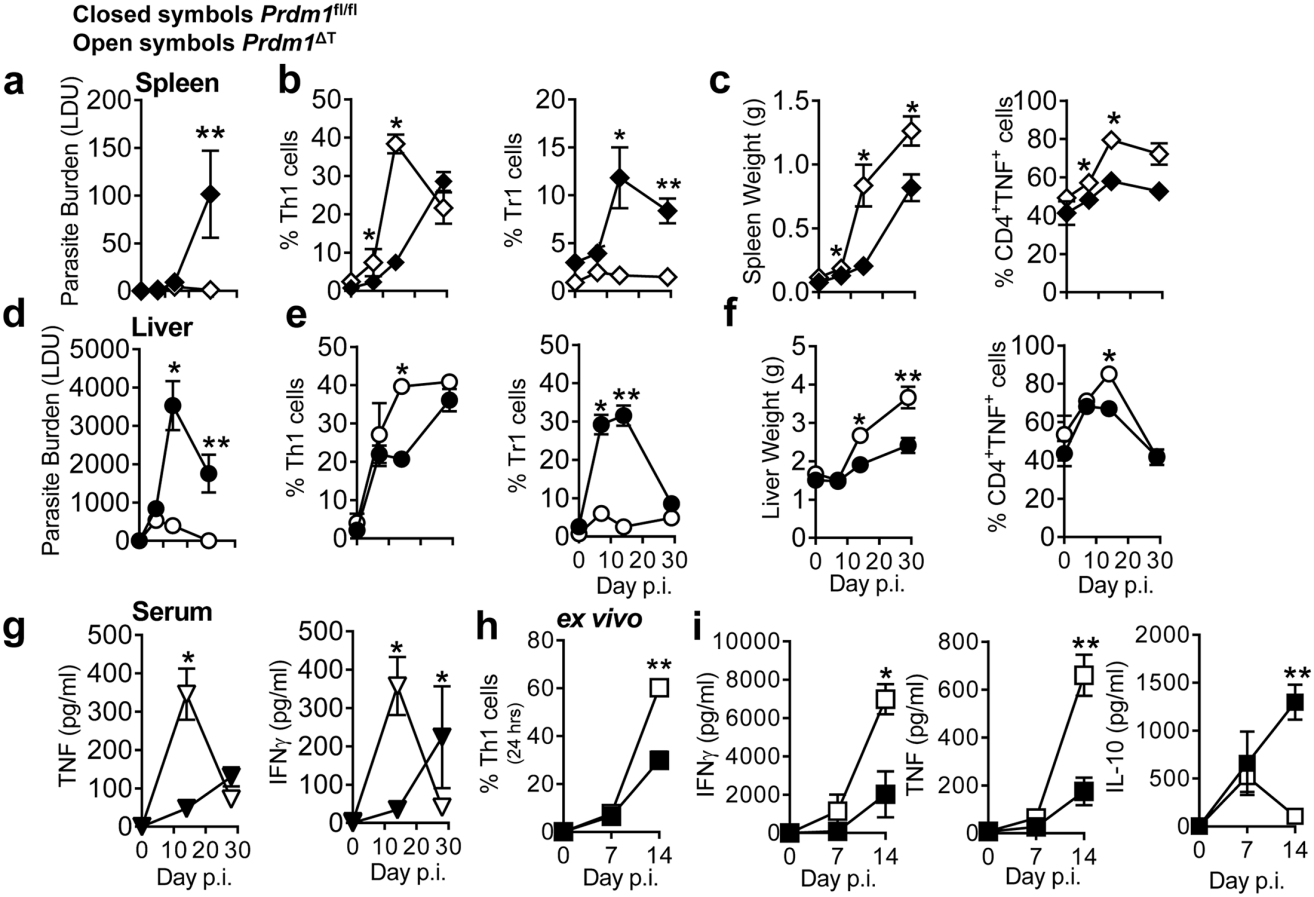
Blimp-1 inhibits the control of parasite growth during experimental visceral leishmaniasis. *Prdm1* ^fl/fl^ and *Prdm1* ^ΔT^ C57BL/6 mice were infected with *L*. *donovani* and spleen (**A**) and liver (**D**) parasite burdens were measured at times indicated. The frequency of Th1 and Tr1 cells in the spleen (**B**) and liver (**E)** were measured by flow cytometry. Organ weights and frequencies of TNF producing CD4^+^ T cells in the spleen (**C**) and liver (**F)** were also assessed, as were serum TNF and IFNγ levels (**G**). Antigen-specific Th1 cell frequency (**H**), IFNγ, TNF and IL-10 production (**I**) were measured in splenocytes after 24 hours of culture in the presence of parasite antigen at times indicated. In all panels, closed shapes represent *Prdm1* ^fl/fl^ mice, while open shapes indicate *Prdm1* ^ΔT^ littermates. Representative of 5 similar experiments, mean ±SEM, n = 5–6 in each group in each experiment, **p<0.01, *p<0.05, Mann-Whitney U test.

**Fig 5:**
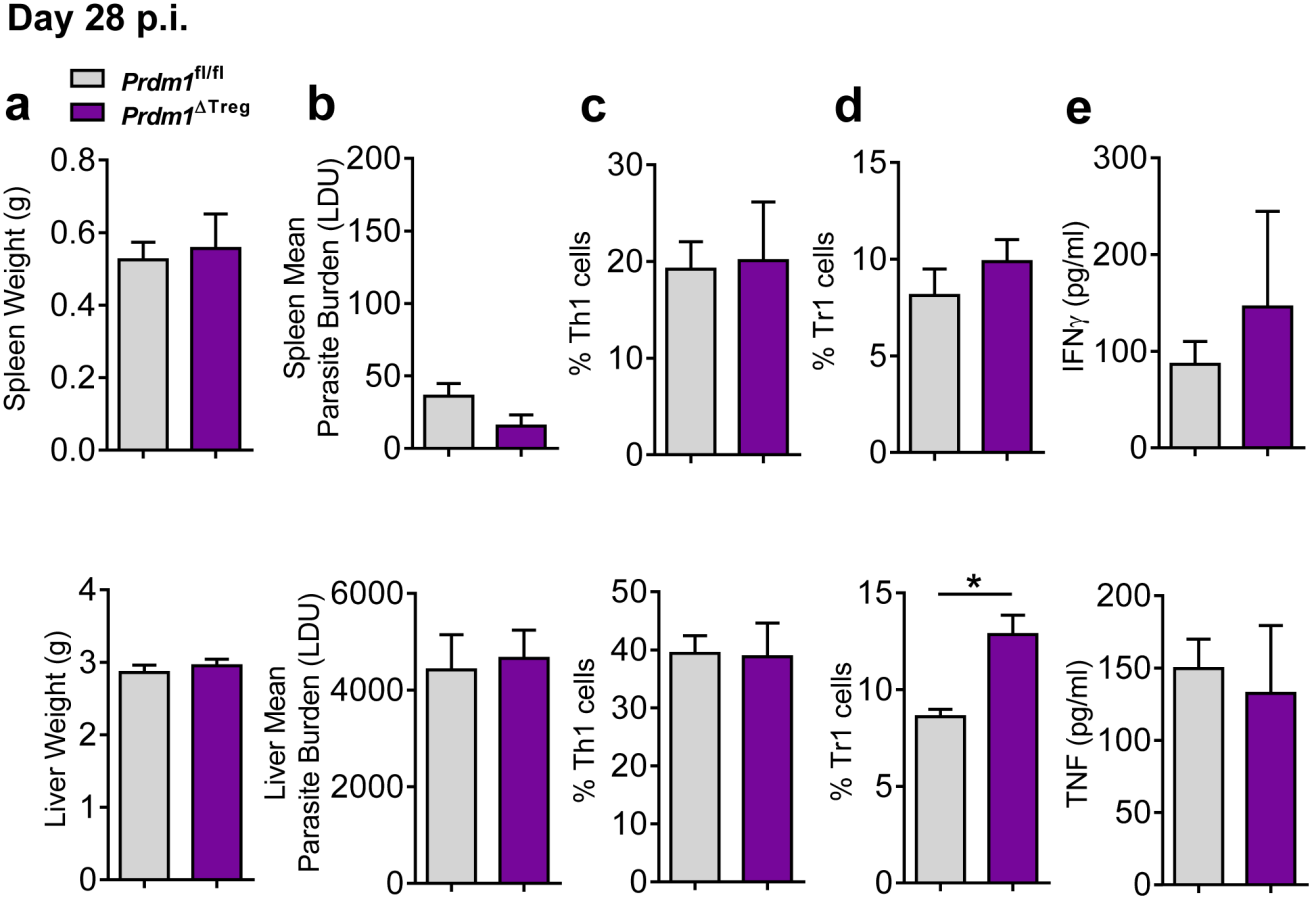
Blimp-1 expression by Treg cells has little influence over host immune responses and parasite growth during experimental visceral leishmaniasis. *Prdm1* ^fl/fl^ and *Prdm1* ^ΔTreg^ C57BL/6 mice were infected with *L*.*donovani*. Spleen and liver weights (**A**), parasite burdens (**B**), frequency of Th1 (**C**) and Tr1 (**D**) cells, as well as serum TNF and IFNγ levels (**E**) were measured at day 28 p.i.. Experiment performed once, mean ±SEM, n = 5, *p<0.05, Mann-Whitney U test.

VL caused by *L*. *donovani* in mice is characterized by a chronic infection of macrophages in the spleen, but acute infection of macrophages in the liver [57]. Strikingly, and in contrast to littermate controls, mice lacking Blimp-1 expression in T cells controlled infection in the spleen effectively (Fig 4A). This improved control of parasite growth was again associated with an increased frequency of activated CD4^+^ T cells and Th1 cells, but restricted development of Tr1 cells (Fig 4B). However, despite improved control of parasite growth in mice with Blimp-1 deficient T cells, these mice presented with significantly larger spleens, associated with an increased frequency of TNF-producing CD4^+^ T cells (Fig 4C). Similar effects were also observed in the liver (Fig 4D–4F), and these were also associated with elevated serum TNF and IFNγ levels (Fig 4G). Enhanced CD4^+^ T cell responses were antigen-specific, as shown by increased IFNγ and TNF production in response to stimulation with parasite antigen (Fig 4H and 4I). Although IL-10 was not detected in serum, IL-10 production was measured in response to antigen re-stimulation, and consistent with the above Tr1 data, was significantly reduced in cells from mice lacking Blimp-1 expression in T cells, compared with littermate controls (Fig 4I). Blimp-1 has previously been shown to restrain CD4^+^ T cell IL-17 production [58], but we found no significant changes in serum IL-17 levels (S5A Fig) or parasite-specific IL-17 production (S5B Fig) in mice with Blimp1-deficient T cells. In addition, *L*. *donovani*-infected mice lacking Blimp-1 expression in FoxP3^+^ T (Treg) cells (*Prdm1* ^ΔTreg^ mice) showed none of the above changes (Fig 5). Thus, lack of Blimp-1 expression by conventional CD4^+^ T cells dramatically improved anti-parasitic immunity, but also promoted tissue pathology. We also observed relatively minor changes in the myeloid cell compartments of mice with Blimp-1-deficient T cells, relative to littermate controls (S6 Fig), most notably, an increased frequency of DC’s in the spleen at day 7 p.i., but decreased frequency in the liver.

### Blimp-1-dependent IL-10 signaling to macrophages protects from tissue pathology

**Fig 6:**
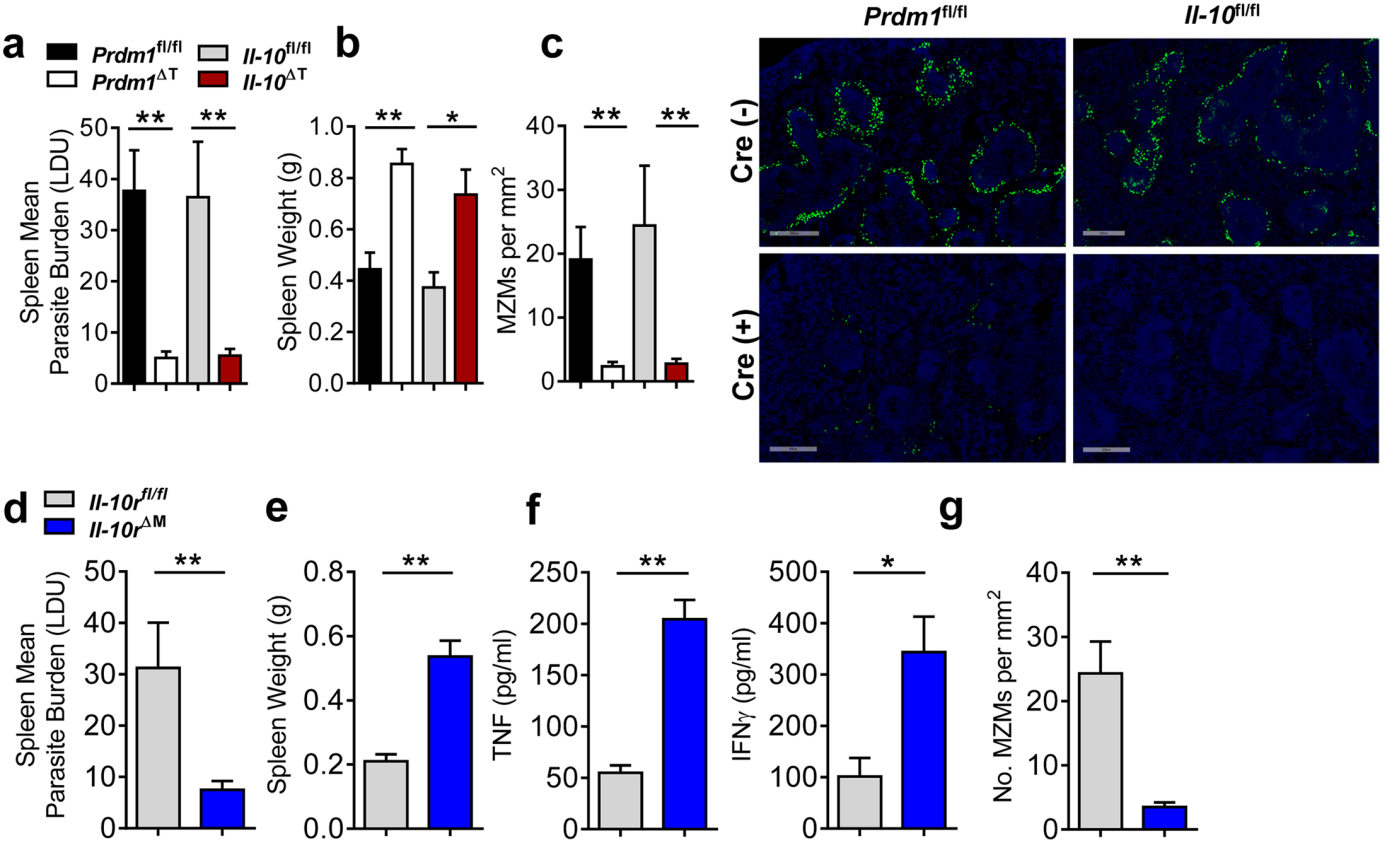
IL-10 signaling to myeloid cells protects macrophages from TNF-mediated destruction. *Prdm1* ^fl/fl^, *Il-10* ^*fl/fl*^, *Prdm1* ^*ΔT*^ and *Il-10* ^*ΔT*^ C57BL/6 mice were infected with *L*. *donovani*. Spleen parasite burdens were determined (**A**) and organ weights were measured (**B**) at day 14 p.i. The number of MZMs per mm^2^ of spleen tissue was determined (**C**), as described in the Materials and Methods. Representative images showing nucleated cells (blue; DAPI) and MZM (indicated by uptake of FITC-dextran, green) (**C**) (objective 20x, scale bars 500 μm). *Il-10r* ^*fl/fl*^ and *Il-10r* ^*ΔM*^ C57BL/6 mice were infected with *L*. *donovani* and spleen parasite burdens (**D**) and spleen weights were measured (**E**) at day 14 p.i., as was serum TNF and IFNγ levels (**F**). The number of MZMs per mm^2^ of spleen tissue was also determined at day 14 p.i. (**G**). Representative of 3 similar experiments, mean ±SEM, n = 5–7 in each group in each experiment, **p<0.01, *p<0.05, Mann-Whitney U test.

To test whether Blimp-1-dependent IL-10 production by T cells was responsible for inefficient control of parasite growth, we infected *Il-10* ^*fl/fl*^ x *Lck-Cre* (*Il-10* ^ΔT^) mice [59] that lacked IL-10 production by T cells with *L*. *donovani*. Similar to mice lacking Blimp-1 expression in T cells, control of parasite growth was dramatically improved, relative to Cre-negative (*Il-10* ^*fl/fl*^) litter mate controls (Fig 6A). As indicated above, the improved parasite clearance in the absence of Blimp-1 in T cells was associated with more severe splenomegaly. IL-10 has previously been found to protect against tissue damage caused by parasite-mediated inflammation [23,24,60], and we show that in *L*. *donovani*-infected mice, increased spleen size was a consequence of a lack of IL-10 production by T cells (Fig 6B). Splenomegaly in mice lacking either Blimp-1 or IL-10 expression in T cells was also associated with a dramatic loss of splenic marginal zone macrophages (MZM) by day 14 p.i. (Fig 6C). Direct IL-10 signaling to myeloid cells contributed to the above phenotypes because mice lacking IL-10 receptor (IL-10R) expression specifically on these cells (*IL-10R* ^*fl/fl*^ x *LysM-Cre* (*Il-10r* ^ΔM^)) [61] and infected with *L*. *donovani* displayed dramatically improved control of parasite growth, which was again accompanied by splenomegaly, increased TNF and IFNγ production, and accelerated loss of MZM, relative to Cre-negative (*Il-10r* ^*fl/fl*^) litter mate controls (Fig 6D–6G). Therefore, Blimp-1-dependent IL-10 produced by CD4^+^ T cells acts on myeloid cells, including MZM, to impair parasite killing, but also acts to limit splenomegaly.

### IL-10 signaling to macrophages protects them from TNF-mediated destruction

**Fig 7:**
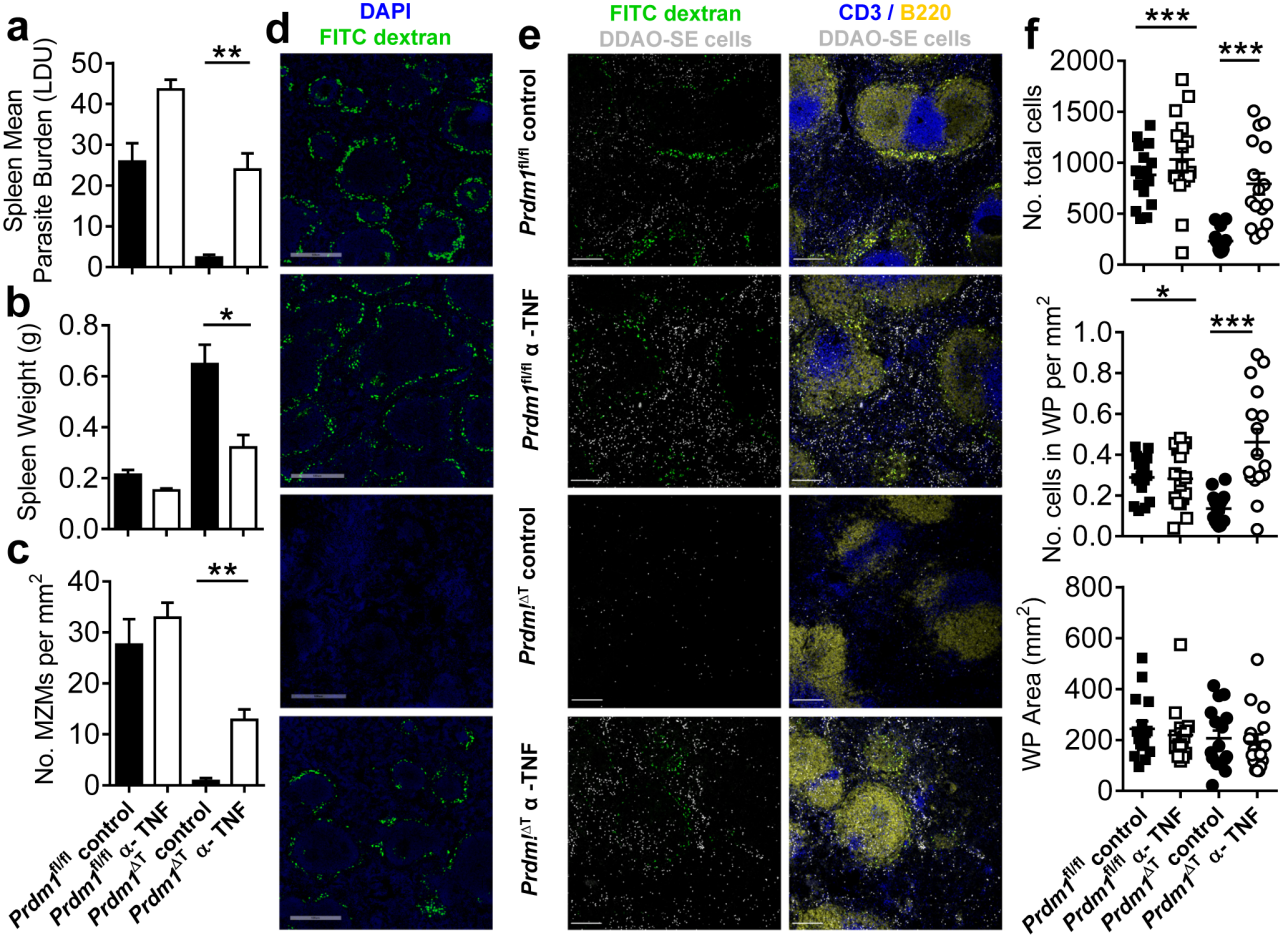
Early loss of marginal zone macrophages and impaired lymphocyte trafficking is a TNF dependent process. *Prdm1* ^fl/fl^ and *Prdm1* ^ΔT^ C57BL/6 mice were infected with *L*. *donovani* and received either TNF blockade (Enbrel) or control human IgG (INTRAGAM), as indicated. Spleen parasite burdens (**A**) and organ weights (**B**) were measured at day 14 p.i., as was the number of MZMs per mm^2^ of spleen tissue (**C**), following labelling of spleen tissue sections for nucleated cells (blue; DAPI) and MZM (indicated by uptake of FITC-dextran, green) (**D**). To examine lymphocyte trafficking, two hours prior to tissue collection, mice were injected with 2 x 10^7^ fluorescently labelled splenocytes. Spleen tissue sections were examined (**E**) for transferred splenocytes (white; left and right panels), T (CD3, blue; right panels) and B (B220, yellow; right panels) cell zones (10x magnification, scale bars = 200 μm). The total number of transferred cells in a given area of spleen tissue, the number of transferred cells per mm^2^ of white pulp (WP) area and total area of WP were calculated as described in Material and Methods (**F**). For each mouse, four fields of view that were identical in size were imaged. Representative of 3 similar experiments, mean ±SEM, n = 5–6 in each group in each experiment, ***p<0.001, **p<0.01, *p<0.05, One-way ANOVA (Kruskal-Wallis test).

We previously showed that following the establishment of chronic *L*. *donovani* infection, TNF mediates the loss of MZM in the spleen, associated with severe disruption of lymphocyte trafficking [51]. Indeed, the accelerated development of splenomegaly and loss of MZM in mice lacking Blimp-1 expression in T cells was associated with disrupted cell trafficking into the spleen, which could be rescued by TNF blockade (Fig 7B–7E). Specifically, MZM were retained following TNF blockade, and this was associated with improved retention of injected, fluorescently-labelled lymphocytes in the T and B cell zones of the white pulp regions of the spleen (Fig 7E and 7F). Interestingly, despite the loss of MZM in mice lacking Blimp-1 expression in T cells, T and B cell zones were largely preserved at day 14 p.i. (Fig 7E and 7F). Importantly, improved parasite control in the absence of Blimp-1 in T cells was also dependent on TNF (Fig 7A), indicating that both beneficial and pathogenic effects of TNF were controlled by Blimp-1-regulated IL-10 production by T cells. While these data demonstrate that TNF blockade can prevent tissue damage during infection, they also highlight the importance of TNF for controlling parasite growth. However, in accordance with previous results [62], established infection could be controlled and anti-parasitic immunity maintained when TNF was blocked when mice were also treated with anti-parasitic drug (S7 Fig), thereby identifying a strategy for controlling TNF-mediated pathology while also controlling parasite growth.

### IFNγ signaling promotes TNF production and associated tissue damage

**Fig 8:**
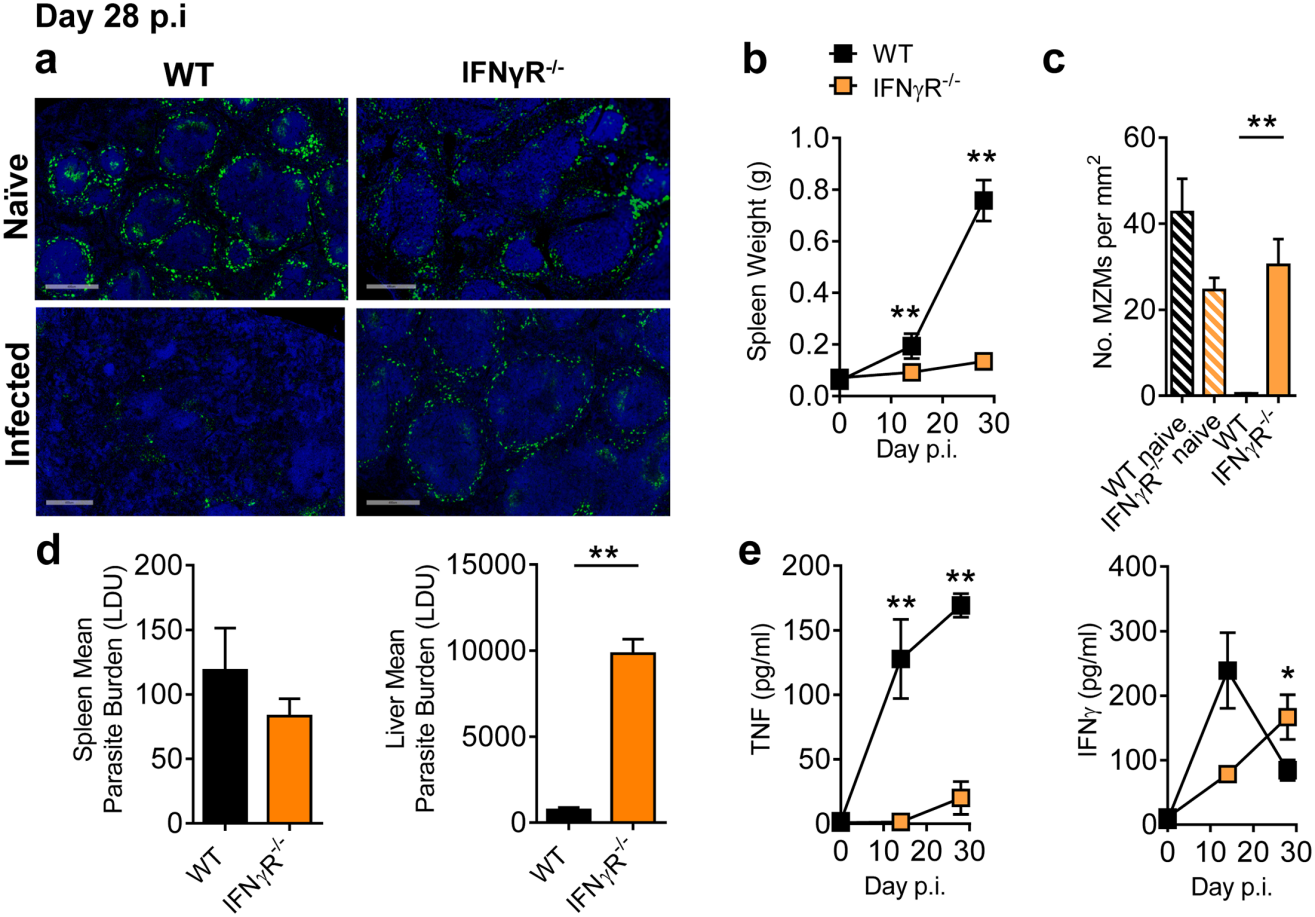
IFNγ signalling drives TNF production and contributes to splenic pathology. C57BL/6 IFNγR^-/-^ and WT mice were infected with *L*.*donovani*. (**A**) Representative images of MZMs at day 28 p.i. (objective 20x, scale bars 500 μm; staining as in Fig 4D). Spleen weights were measured at times indicated (**B**) and the number of MZMs per mm^2^ of spleen tissue was determined at day 28 p.i. (**C**), as described in the Materials and Methods. Spleen and liver parasite burdens were measured (**D**) at day 28 p.i. as was serum TNF and IFNγ levels (**E**) Representative of 3 similar experiments, mean ±SEM, n = 6 in each group in each experiment, **p<0.01, *p<0.05, Mann-Whitney U test.

As outlined above, improved parasite control and exacerbated tissue pathology in mice lacking Blimp-1 or IL-10 expression in their T cells was strongly associated with increased numbers of Th1 cells and serum IFNγ levels (Fig 4). We therefore examined how IFNγ production influenced TNF-mediated consequences of *L*. *donovani* infection described above. Strikingly, despite uncontrolled hepatic parasite growth, as reported in IFNγ-deficient mice [63], mice lacking IFNγ receptor (IFNγR) showed no splenomegaly and complete preservation of MZM after 28 days of infection when MZM were lost in C57BL/6 controls (Fig 8A–8D). Critically, these mice produced minimal amounts of TNF, relative to wild type controls (Fig 8E). Thus, our results show that following *L*. *donovani* infection, IFNγ promoted TNF production, and this pathway was regulated by Blimp-1-mediated IL-10 production by T cells. Importantly, this regulatory pathway determined the balance between control of parasite growth and TNF-mediated pathology.

## Discussion

Here we show that Blimp-1 mRNA was elevated in CD4^+^ T cells from VL patients, along with IL-10 mRNA and elevated levels of plasma IL-10. Furthermore, in an experimental model of VL, Blimp-1-dependent IL-10 produced by Tr1 cells acted on myeloid cells to limit parasite killing, but was critical to prevent TNF-mediated tissue disruption. In the absence of IL-10 production by T cells, MZM were lost and this was associated with disrupted lymphocyte trafficking and splenomegaly. Thus, we have identified Tr1 cells as potent suppressors of anti-parasitic immunity, but critical regulators of IFNγ-dependent, TNF-mediated pathology.

The transcriptional regulator Blimp-1 is important for IL-10 production by both Treg [64] and Tr1 [52,53] cells. However, Blimp-1 deficiency in Treg cells has minimal impact on immune responses and disease outcome in mice infected with *L*. *donovani*. Data from malaria [30,31] and VL [29] patients indicates that Tr1 cells are a major regulatory T cell population during protozoan diseases, and our results show a critical role for Blimp-1 in Tr1 cell function by promoting IL-10 production. Furthermore, results from mice that lack IL-10 in the T cell compartment indicate that IL-10 is a critical immune mediator being controlled by Blimp-1 following *L*. *donovani* infection. Another striking feature in mice lacking Blimp-1 expression in T cells was the increase in number and frequency of activated CD4^+^ T cells. Earlier work showed that Blimp-1-deficient T cells do not have an intrinsically better ability to proliferate [65,66], but they are more resistant to activation-induced cell death [65]. In addition, Blimp1 is an important repressor of T follicular helper (Tfh) cell development by suppressing Bcl6 [67]. Thus, one potential explanation for increased CD4^+^ T cell activation in our disease models is less cell death, as well as cell differentiation favoring Th1 cell development.

The control of many intracellular infections requires Th1 cells which are generated in response to macrophage and dendritic cell derived IL-12 [9]. These Th1 cells produce IFNγ and TNF to activate phagocytes and kill intracellular pathogens [10]. However, if pathogens persist, there is a danger that these pro-inflammatory cytokines will damage tissue, and consequently, Tr1 cells develop to control this inflammation [12,33–35]. Our results suggest that Tr1 cells generated in our experimental setting derive from Th1 cells, a notion supported by recent results showing IL-12, along with IL-27, promoted Blimp-1-dependent IL-10 production by Tr1 cells [52]. Thus, our data suggest that IL-12 is not only required to generate Th1 cells following *L*. *donovani* infection, but also provides additional signals for the transition from Th1 to Tr1 cells in this model. Previous work by others has already identified an important role for IL-27 in Tr1 cell development in experimental malaria [23,68] and leishmaniasis [56,69].

TNF is involved in the pathogenesis of a range of diseases, including infectious and autoimmune diseases, and more recently, complications arising in immune-related adverse events as a consequence of immune check point inhibition [3,70]. Our results identify TNF as a major mediator of tissue pathology in the absence of T cell-derived IL-10. Hence, understanding how TNF is regulated in different disease settings is of major medical importance. Despite differences in the structure of rodent and human spleens [38], post-mortem studies on VL patients revealed extensive disruption to white pulp areas, associated with substantial changes in macrophage populations [47,48], also reported in experimental VL [50,51]. The MZ of the spleen is a specialized collection of cells separating the predominantly non-lymphoid red pulp regions and lymphoid dominated white pulp regions. It is also vascular and plays an important role in removing particulate antigen, as well as dead and dying cells, from the circulation [38,71]. Remarkably, the accelerated loss of MZM in mice lacking Blimp-1 or IL-10 expression in T cells resulted in significant disruption of lymphocyte trafficking into T and B cell zones of the white pulp that could be rescued by TNF blockade. These results support earlier studies showing important roles for MZM in directing lymphocyte traffic into the splenic white pulp [72,73]. Another striking feature of *L*. *donovani*-infected mice lacking Blimp-1 or IL-10 expression by T cells was dramatic splenomegaly. Angiogenesis is a dominant feature of splenomegaly during experimental VL [44], driven by inflammation-induced expression of neurotropic receptor on vascular endothelium and interactions with ligands produced by mononuclear phagocytes [43]. TNF produced by macrophages can promote angiogenesis [74,75], and chronic inflammation and vascular remodelling are intimately linked in autoimmune disease settings [76]. Critically, inhibition of vascularization with receptor tyrosine kinase inhibitors in mice with established *L*. *donovani* infection resulted in reduced mononuclear phagocyte number and reversed splenomegaly [44]. Therefore, our data supports a model whereby TNF-driven angiogenesis is regulated by Blimp-1-dependent IL-10 production by Tr1 cells.

By identifying IL-10 produced by Tr1 cells as critical regulators of TNF, we can consider IL-10-related strategies for modulating TNF production. However, given the important role of TNF in controlling intracellular infections [62,77], these strategies should be designed to limit pathogenic functions of TNF while maintaining anti-microbial effects. TNF is produced by many different cell populations, and acts on a range of target cells [78]. We previously reported that TNF from CD4^+^ T cells was required for anti-parasitic functions in *L*. *donovani*-infected mice [77], but the cellular source of pathogenic TNF is not known. In ulcerative colitis patients, TNF from T cells appeared to be an important driver of disease [79], while in graft versus host disease, both T cell [80] and macrophage/monocyte-derived TNF cause gastrointestinal damage [81]. Therefore, different cellular sources of TNF are likely to be important for pathogen control and promoting disease in different immune environments. Further work is needed to better understand the cellular and molecular aspects of TNF regulation that will allow selective targeting of these two distinct functional outcomes of TNF biology.

Treg cells are currently being generated and tested for a range of inflammatory conditions [82,83]. However, in situations where this approach fails, the use of Tr1 cells may be beneficial [84]. Our data indicate that Tr1 cells play a critical role in regulating inflammation in organs such as the spleen, in contrast to inflammation in the lung or gut, where Treg cells play critical protective roles [18,85,86]. Hence, under different clinical situations, either Treg or Tr1 cells may help to treat disease. Our findings identify Blimp-1-dependent IL-10 produced by Tr1 cells as a critical regulator of IFNγ-dependent, TNF-mediated tissue damage in the spleen in parasitic infections. Thus, Tr1 focused therapy may be an attractive modality in settings where TNF-mediated immunity is needed, but TNF-induced immunopathology instead dominates.

## Methods

### Ethics statement

All patients presented with symptoms of VL at the Kala-azar Medical Research Center (Muzaffarpur, Bihar, India). VL diagnosis was confirmed either by the microscopic detection of amastigotes in splenic aspirate smears or by rk39 dipstick test. Patients were treated either with Amphotericin B or Ambisome. In total, 10 patients were enrolled in the study. The use of human subjects followed recommendations outlined in the Helsinki declaration. Written informed consent was obtained from all participants and/or their legal guardian when under 18 years of age. Ethical approval (Dean/2011-12/289) was obtained from the ethical review board of Banaras Hindu University (BHU), Varanasi, India.

All animal procedures were approved by the QIMR Berghofer Medical Research Institute Animal Ethics Committee. This work was conducted under QIMR Berghofer animal ethics approval number A02-634M, in accordance with the “Australian Code of Practice for the Care and Use of Animals for Scientific Purposes” (Australian National Health and Medical Research Council).

### Mice

Female C57BL/6J mice, 8–12 weeks old were purchased from the Australian Resource Centre (Canning Vale, WA, Australia) and the Walter and Eliza Hall Institute (Melbourne, VIC, Australia). *Prdm1* ^*fl/fl*^, *Prdm1* ^ΔT^, *Prdm1* ^*GFP/+*^ *Il-10* ^*fl/fl*^, *Il-10* ^*ΔT*^, *Il-10r* ^*fl/fl*^ and *Il-10r* ^*ΔM*^ mice were bred in-house under specific-pathogen free conditions. *Prdm1* ^ΔTreg^ mice were bred at the Walter and Eliza Hall Institute. All *Prdm1* ^*fl/fl*^ [54] and *Prdm1* ^*GFP/+*^ [55] mice were on a pure C57BL/6 background, while *Il-10* ^*fl/fl*^ [59] and *Il-10r* ^*fl/fl*^ [61] mice were backcrossed to C57BL/6 for at least 10 generations.

### Parasites and infections

*Leishmania donovani* (LV9) parasites were maintained by passage in B6.*Rag1* ^-/-^ mice and amastigotes were isolated from the spleens of chronically infected mice. Mice were infected with 2 x 10^7^ LV9 amastigotes intravenously (i.v.) via the lateral tail vein. Spleen and liver impression smears were used to determine parasite burdens and were expressed as Leishman Donovan Units (LDU; number of amastigotes per 1000 host nuclei multiplied by the organ weight (in grams)).

*Plasmodium chabaudi chabaudi* AS (PcAS) and *Plasmodium berghei* ANKA (PbA) strains were used in all experiments after one *in vivo* passage in a C57BL/6 mouse. All mice received a dose of 10^5^ pRBCs i.v. via the lateral tail vein. Thin blood smears from tail bleeds were stained with Clini Pure- stains (HD Scientific Supplies, Willawong, Australia). Parasitemia was used to monitor the course of infection and was determined by flow cytometry (see below).

### Monitoring parasitemia by flow cytometry during experimental malaria

Briefly, 1–2 drops of blood from a tail bleed was diluted and mixed in 250 μl RPMI/PS containing 5 U/ml heparin sulphate. Diluted blood was stained simultaneously with Syto84 (5 μM; Life Technologies, Mulgrave, Australia) to detect RNA/DNA and Hoechst33342 (10 μg/ml; Sigma-Aldrich, Castle Hill, Australia) to detect DNA for 30 minutes at room temperature, protected from light. 2 ml RPMI/PS was added to stop the reaction, and samples were immediately placed on ice until acquisition on a BD FACSCanto II Analyzer (BD Biosciences, Franklin Lakes, NJ). Data was analysed using FlowJo software (Treestar), where pRBC were readily detected as being Hoechst33342^+^ Syto84^+^, with lymphocytes excluded on the basis of size, granularity, and higher levels of Hoechst33342/Syto84 staining compared with pRBCs.

### Human VL patient samples

Heparinized blood was collected from patients before and 28 days after commencement of drug treatment, and PBMC were isolated by Ficoll-Hypaque (GE Healthcare, NJ) gradient centrifugation and used for the positive selection of CD4^+^ T cells using magnetic beads and columns (Miltenyi Biotech, Bergisch Gladbach, Germany). Cells were collected directly into RNAlater (Sigma), and stored at -70°C until mRNA isolation and analysis. Total RNA was isolated using RNeasy mini kits and QiaShredder homogenizers (Qiagen, Valencia, CA), according to the manufacturer’s protocol. The quality of RNA was assessed by denaturing agarose gel electrophoresis. cDNA synthesis was performed in 20 μL reactions on 0.5–1.0 μg RNA using High-Capacity cDNA Archive kit (Applied Biosystems, CA, USA). Real-time PCR was performed on an ABI Prism 7500 sequence detection system (Applied Biosystems) using cDNA-specific FAM–MGB labelled primer/probe for *PRDM1*. The relative quantification of products was determined by the number of cycles over 18S mRNA endogenous control required to detect *PRDM1* gene expression.

### *L*. *donovani* antigen re-stimulation assay

Spleens were processed through a 100μm cell strainer in order to obtain a single-cell suspension. Splenocyte cell suspensions were then counted and adjusted to a concentration of 2 x 10^6^ cells/ml. LV9 amastigotes (fixed in 4% PFA) were thawed and washed in RPMI media containing Penincillin-Streptomycin and then counted and adjusted to a final concentration of 4 x 10^7^/ml. Cells and parasites were plated into a 96-U well plate at a 1:20 ratio, where each well contained 1 x 10^5^ cells and 2 x 10^6^ parasites. Cells were cultured in the presence of antigen for a period of 24 and 72 hours. Culture supernatants were harvested at 24 and 72 hours and intracellular cytokine staining was performed at both time points.

### Monitoring *PbA* infection and clinical scoring of ECM symptoms

A transgenic *PbA* line (231c11) expressing luciferase (*PbA*-luc) and GFP under the control of the ef1-α promoter [87] was used for all *PbA* experiments. *PbA*-infected mice were monitored and scored, as previously described [88].

### *In vivo* bioluminescence imaging

The *in vivo* imaging system 100 (Xenogen, Alameda, CA) was used to detect the level of bioluminescence as a measure of whole body parasite burden in each mouse. At selected time-points, *PbA*-luc-infected mice were anaesthetised with isofluorane and injected with 150 mg/kg i.p. of D-luciferin (Xenogen) 5 minutes prior to imaging. Bioluminescence was measured in p/s/cm²/sr using Living Image (Xenogen), as previously described [88].

### Antibody treatment

For IL-12 neutralisation experiments, mice were administered 500 μg of rat IgG (Sigma) or anti-IL-12 (clone: C17.8; BioXcell; West Lebanon, NH) i.p., on the day of infection and every 3 days post infection until day 14 p.i. For TNF blockade experiments, mice administered 200 μg of Human Normal Immunoglobulin (INTRAGAM P; CSL, Melbourne, Australia) or anti-TNF (Enbrel; Amgen, Thousand Oaks, CA) i.p., on the day of infection and every 2 days post infection until day 14 p.i..

### Fluorescence microscopy

Mice were injected with 100 μg i.v. of FITC dextran (Life Technologies, Melbourne, Australia) one day prior to collection of organs. Spleen tissue was collected into 4% PFA, incubated at room temperature for 1-2hrs and then transferred to a 30% sucrose solution (in MilliQ water) (Sigma, Sydney, Australia) overnight at 4°C. Fixed spleen tissue was then preserved in Tissue-Tek O.C.T. compound (Sakura, Torrance, CA). Splenic architecture and distribution of marginal zone macrophages (MZMs) were analysed in 20 μm sections counter-stained with DAPI and visualized on the Aperio FL slide scanner. Image analysis was performed using Image Scope to determine area of the sections and Metamorph 7.8 (Integrated Morphometry analysis tool; Molecular Devices, Sunnyvale, CA) to count the MZMs. In some experiments, mice were injected intravenously with 2 x 10^7^ naïve splenocytes labelled with Cell Trace Far Red DDAO-SE (Life Technologies, Mulgrave, Australia), 2 hours prior to sacrifice. The same 20 μm sections were used to assess cell trafficking, where sections were stained with CD3 biotin (5 μg/ml) + SA AF594 (5 μg/ml), B220 PE (5 μg/ml) (Biolegend, San Diego, CA), counter-stained with DAPI (1:25000, Sigma-Aldrich, Castle Hill, Australia) and mounted with Pro-Long Gold anti-fade (Life Technologies). Slides were visualized on a Carl Zeiss 780 NLO laser scanning confocal microscope under 10x magnification. Image analysis was performed using Metamorph 7.8 (Counting App and Region Measurement tool). Briefly, since each image was identical in size (1416.30μm x 1416.30μm), the total number of cells was counted in each image. The drawing tool was used to delineate the T and B cell zones in the white pulp (WP) of the spleen and the area of these zones in each image was measured (in mm^2^). Number of cells in WP per mm^2^ was calculated as the cumulative number of cells in WP in each image divided by cumulative areas of WP in each image.

### Flow cytometry

Allophycocyanin-conjugated anti–IFNγ (XMG1.2), anti-IL-10 (JES5-16E3), PE-conjugated TNFα (MP6-XT22) allophycocyanin-Cy7–conjugated anti-CD4 (GK1.5), FITC-conjugated anti-CD11a (M17/4), Brilliant Violet 421–conjugated anti–IFNy (clone XMG1.2), allophycocyanin-Cy7–conjugated anti-NK1.1 (clone PK136), Alexa Fluor 700–conjugated anti-CD8a (clone 53–6.7), biotinylated-CD49d (R1-2), PeCy7-conjugated Strepavidin and PerCP-Cy5.5–conjugated anti–TCRb-chain (H57-597), Allophycocyanin-conjugated anti–anti-CD11c (N418), Pacific Blue-conjugated anti-MHCII (I-A/I-E) (M5/114-15.3), PerCP-Cy5.5–conjugated anti-CD11b (M1/70), FITC-conjugated anti-Ly6C (HK1.4), PeCy7-conjugated anti-F4/80 (BM8) Brilliant Violet 605-conjugated anti-TCRβ (H57-597), allophycocyanin-Cy7–conjugated anti-B220 (RA3-6B2) and Alexa Fluor 700-conjugated Strepavidin were purchased from BioLegend (San Diego, CA) or BD Biosciences. Dead cells were excluded from the analysis using LIVE/DEAD Fixable Aqua Stain or LIVE/DEAD Fixable Near Infra-Red Stain (Invitrogen-Molecular Probes, Carlsbad, CA), according to the manufacturer’s instructions. The staining of cell surface antigens and intracellular cytokine staining were carried out as described previously [89,90]. FACS was performed on a FACSCanto II or LSRFortessa (BD Biosciences), and data was analyzed using FlowJo software (TreeStar). Gating strategies used for analysis are shown in Figs 1 and 2.

### Measurement of serum and culture supernatant cytokine levels

Cytokine levels in the serum and culture supernatants were measured using a BD Cytometric Bead Array (CBA) Flex sets and the HTS system plate reader on the Fortessa 5 Flow cytometer (BD Biosciences) according to the manufacturer’s instructions.

### Statistical analysis

Comparisons between two groups were performed using non-parametric Mann-Whitney tests in mouse studies and Wilcoxon matched-pairs signed rank test in human studies. Comparisons between multiple groups were made using a Kruskal-Wallis test and corrected using Dunn’s multiple comparisons test. GraphPad Prism version 6 for Windows (GraphPad, San Diego, CA) was used for analysis; p<0.05 was considered statistically significant. All data are presented as the mean ± SEM.

## Supporting Information

S1 Fig *Prdm1* ^fl/fl^ and *Prdm1* ^ΔT^ C57BL/6 mice were infected with PcAS and the frequency and number of splenic CD8^+^ T cells expressing IFNγ and Tbet (**A**) and IL-10 and IFNγ (**B**) were measured by flow cytometry at day 15 p.i. Representative of 3 similar experiments, mean ±SEM, n = 5 in each group in each experiment, **p<0.01, *p<0.05, Mann-Whitney U test.
(TIF)

S2 Fig Female C57BL/6 mice were infected with PcAS and the frequency of activated CD4^+^ T cells (CD11a^+^ CD49d^+^) (**A** and **B**), Th1 cells (Tbet^+^ IFNγ^+^) (**C** and **D**) and Tr1 cells (IFNγ^+^ IL-10^+^) (**E** and **F**) were measured at the time points indicated. All Th1 and Tr1 cells were contained within the activated CD4^+^ T cell compartment (**G**).
(TIF)

S3 Fig *Prdm1* ^fl/fl^ and *Prdm1* ^ΔT^ C57BL/6 mice were infected with PcAS and the frequency of activated CD4^+^ T cells (CD11a^+^ CD49d^+^) Th1 cells (Tbet^+^ IFNγ^+^) and Tr1 cells (IFNγ^+^ IL-10^+^) in the spleen were measured at day 4 (**A**) and 7 (**B**) p.i. Representative of 2 similar experiments, mean ±SEM, n = 5 in each group in each experiment, **p<0.01, *p<0.05, Mann-Whitney U test.
(TIF)

S4 Fig *Prdm1* ^fl/fl^ and *Prdm1* ^ΔT^ C57BL/6 mice were infected with *Pb*A-luc and whole body parasite burdens and blood parasitemia (**A**) was measured at day 6 p.i., when *Prdm1* ^fl/fl^ mice first began to exhibit early ECM symptoms. Clinical scores and percent survival (**B**) was determined. Grey area indicates time frame when neurological symptoms were apparent in Cre (-) mice. Dotted line at clinical score 4 indicates moribund threshold. Frequency and numbers of Th1 (**C**) and Tr1 (**D**) cells were assessed by flow cytometry at day 4 p.i. Representative of 3 similar experiments, mean ±SEM, n = 5–6 in each group in each experiment, ***p<0.001, **p<0.01, *p<0.05, Mann-Whitney U test, log-rank (Mantel-Cox) test (percent survival).
(TIF)

S5 Fig *Prdm1* ^fl/fl^ and *Prdm1* ^ΔT^ C57BL/6 mice were infected with *L*. *donovani* and serum IL-17A levels (**A**), as well as antigen-specific IL-17A production by splenocytes were measured after 72 hours of culture in the presence of parasite antigen (**B**) at times indicated. In both panels, closed shapes represent *Prdm1* ^fl/fl^ mice, while open shapes indicate *Prdm1* ^ΔT^ littermates. Representative of 2 similar experiments, mean ±SEM, n = 5–6 in each group in each experiment.
(TIF)

S6 Fig *Prdm1* ^fl/fl^ and *Prdm1* ^ΔT^ C57BL/6 mice were infected with *L*. *donovani* and monocytes, DC’s and neutrophils identified by the gating strategy shown in (**A**) and their frequency measured in the spleen (**B**) and liver (**C**) at time points indicated. In all panels, closed shapes represent *Prdm1* ^fl/fl^ mice, while open shapes indicate *Prdm1* ^ΔT^ littermates. Representative of 2 similar experiments, mean ±SEM, n = 5 in each group in each experiment, **p<0.01, *p<0.05, Mann-Whitney U test.
(TIF)

S7 Fig C57BL/6 mice were infected with *L*. *donovani* and received either TNF blockade (Enbrel) or control human IgG (INTRAGAM), with or without sodium stibogluconate (SSG), as indicated, from days 14–28 p.i.. Liver (**A**) and spleen (**B**) parasite burdens and spleen weights (**C**) were measured at day 28 p.i., as was the number of MZMs per mm^2^ of spleen tissue ((**D)**; as described in Fig 4C). Th1 cell frequency in splenocytes cultured in media or with parasite antigen (**E**), as indicated, as well as IFNγ production from antigen-stimulated cells (**F**) were measured after 24 hours of culture. Representative of 2 independent experiments, mean ±SEM, n = 5, **p<0.01, *p<0.05, Mann-Whitney U test.
(TIF)
