# Correction: Blimp-1-Dependent IL-10 Production by Tr1 Cells Regulates TNF-Mediated Tissue Pathology

**DOI:** 10.1371/journal.ppat.1005460

**Published:** 2016-02-12

**Authors:** Marcela Montes de Oca, Rajiv Kumar, Fabian de Labastida Rivera, Fiona H Amante, Meru Sheel, Rebecca J. Faleiro, Patrick T. Bunn, Shannon E. Best, Lynette Beattie, Susanna S. Ng, Chelsea L. Edwards, Werner Muller, Erika Cretney, Stephen L. Nutt, Mark J. Smyth, Ashraful Haque, Geoffrey R. Hill, Shyam Sundar, Axel Kallies, Christian R. Engwerda

The following information is missing from the Funding section: This study was supported by funding from the National Institute of Allergy and Infectious Diseases, National Institutes of Health Tropical Medicine Research Centre Program, Grant ID No: 2P50AI7434.
