# Supplementary figures and images for: Blimp-1-Dependent IL-10 Production by Tr1 Cells Regulates TNF-Mediated Tissue Pathology
Source: PLoS Pathog. 2016 Jan 14;12(1):e1005398. doi: 10.1371/journal.ppat.1005398 (PMC4713066; doi:10.1371/journal.ppat.1005398)

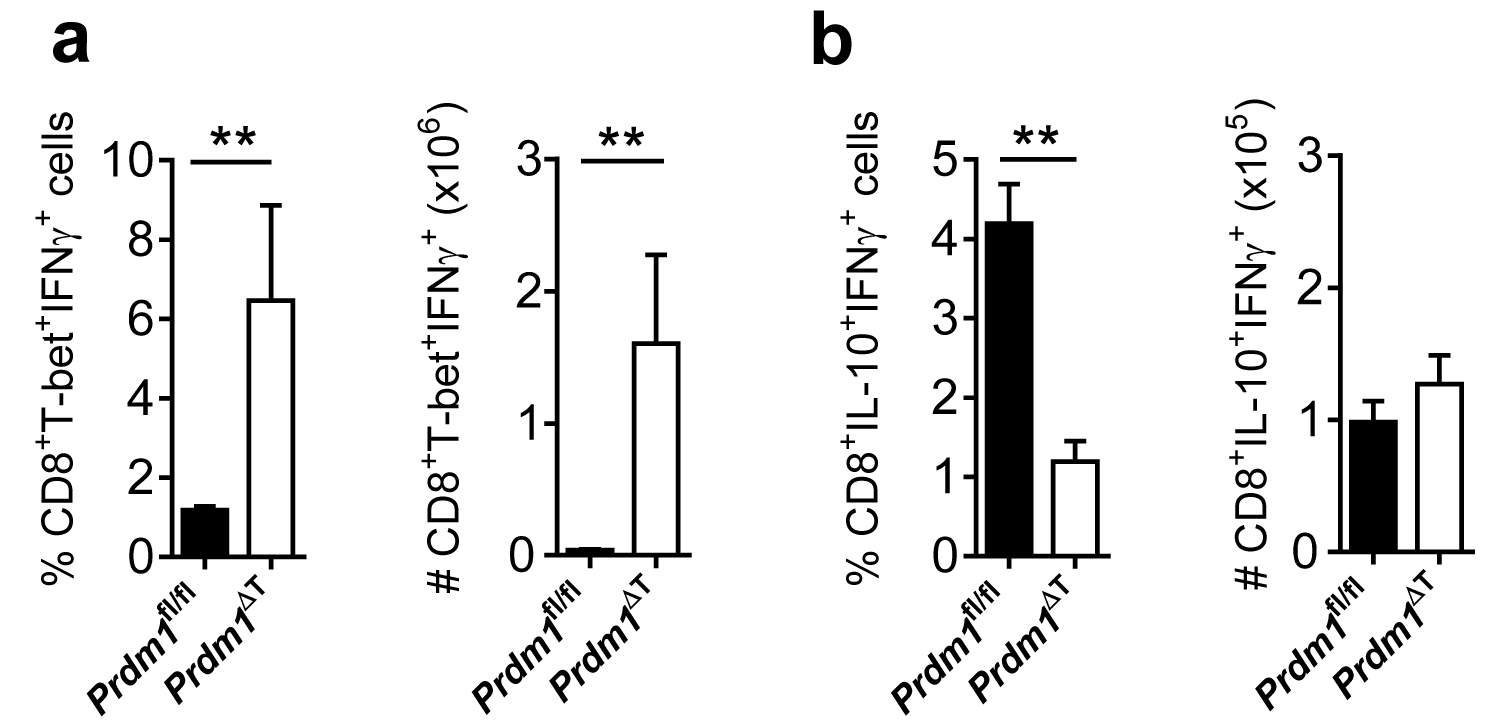

Supplement: S1 Fig — Prdm1 fl/fl and Prdm1 ΔT C57BL/6 mice were infected with PcAS and the frequency and number of splenic CD8+ T cells expressing IFNγ and Tbet (A) and IL-10 and IFNγ (B) were measured by flow cytometry at day 15 p.i. Representative of 3 similar experiments, mean ±SEM, n = 5 in each group in each experiment, **p<0.01, *p<0.05, Mann-Whitney U test. (TIF) [file ppat.1005398.s001.tif]

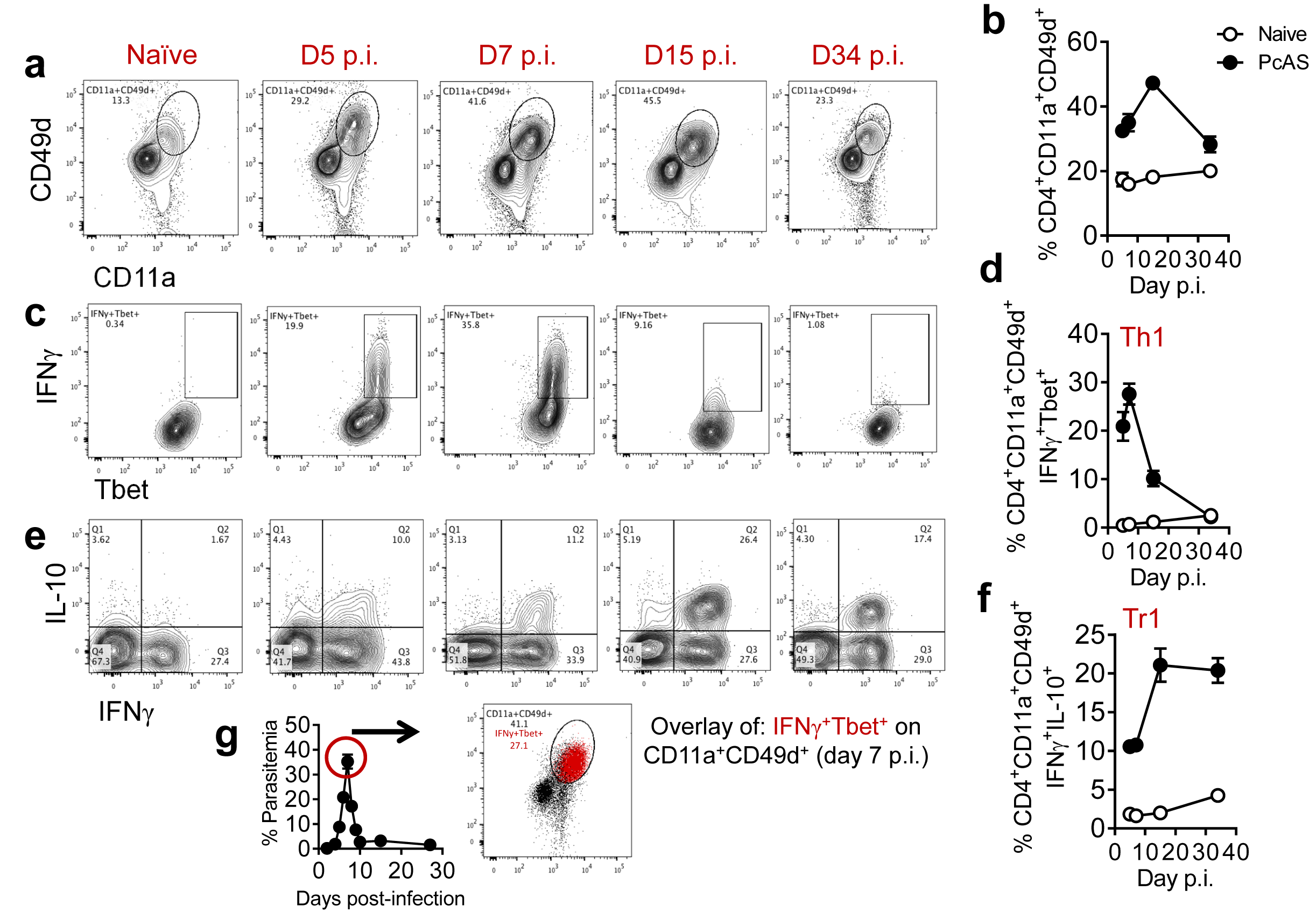

Supplement: S2 Fig — Female C57BL/6 mice were infected with PcAS and the frequency of activated CD4+ T cells (CD11a+ CD49d+) (A and B), Th1 cells (Tbet+ IFNγ+) (C and D) and Tr1 cells (IFNγ+ IL-10+) (E and F) were measured at the time points indicated. All Th1 and Tr1 cells were contained within the activated CD4+ T cell compartment (G). (TIF) [file ppat.1005398.s002.tif]

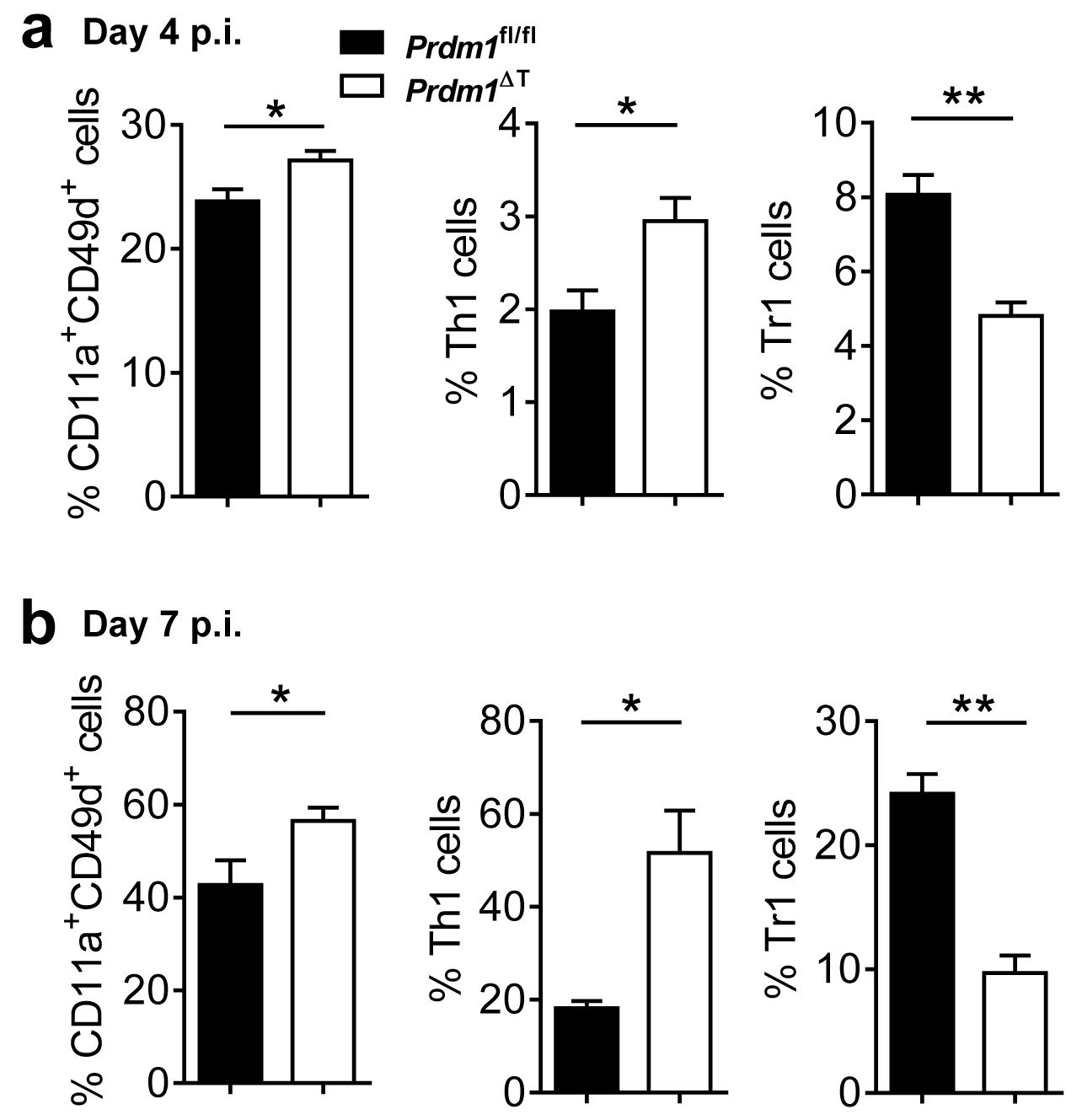

Supplement: S3 Fig — Prdm1 fl/fl and Prdm1 ΔT C57BL/6 mice were infected with PcAS and the frequency of activated CD4+ T cells (CD11a+ CD49d+) Th1 cells (Tbet+ IFNγ+) and Tr1 cells (IFNγ+ IL-10+) in the spleen were measured at day 4 (A) and 7 (B) p.i. Representative of 2 similar experiments, mean ±SEM, n = 5 in each group in each experiment, **p<0.01, *p<0.05, Mann-Whitney U test. (TIF) [file ppat.1005398.s003.tif]

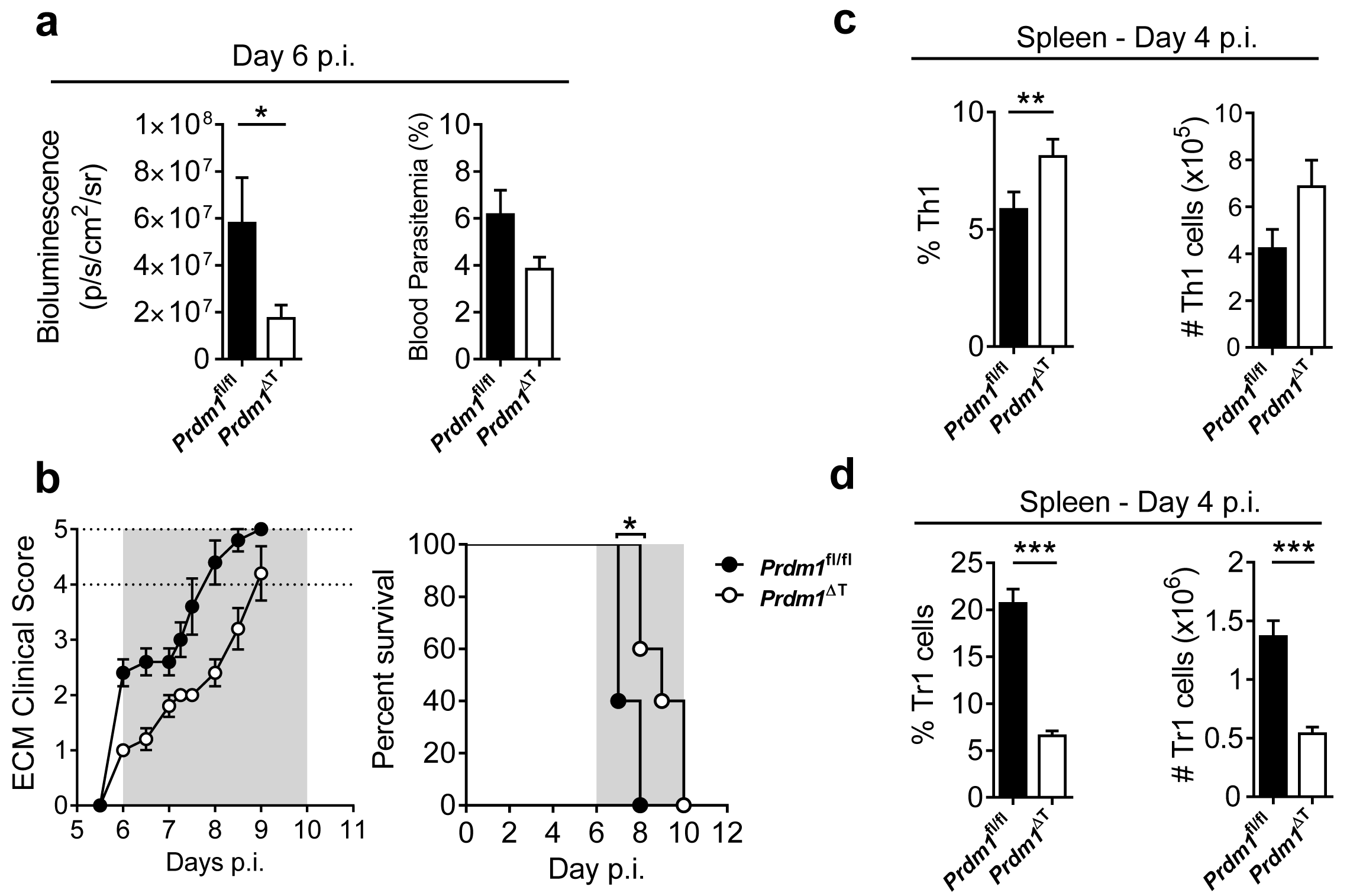

Supplement: S4 Fig — Prdm1 fl/fl and Prdm1 ΔT C57BL/6 mice were infected with PbA-luc and whole body parasite burdens and blood parasitemia (A) was measured at day 6 p.i., when Prdm1 fl/fl mice first began to exhibit early ECM symptoms. Clinical scores and percent survival (B) was determined. Grey area indicates time frame when neurological symptoms were apparent in Cre (-) mice. Dotted line at clinical score 4 indicates moribund threshold. Frequency and numbers of Th1 (C) and Tr1 (D) cells were assessed by flow cytometry at day 4 p.i. Representative of 3 similar experiments, mean ±SEM, n = 5–6 in each group in each experiment, ***p<0.001, **p<0.01, *p<0.05, Mann-Whitney U test, log-rank (Mantel-Cox) test (percent survival). (TIF) [file ppat.1005398.s004.tif]

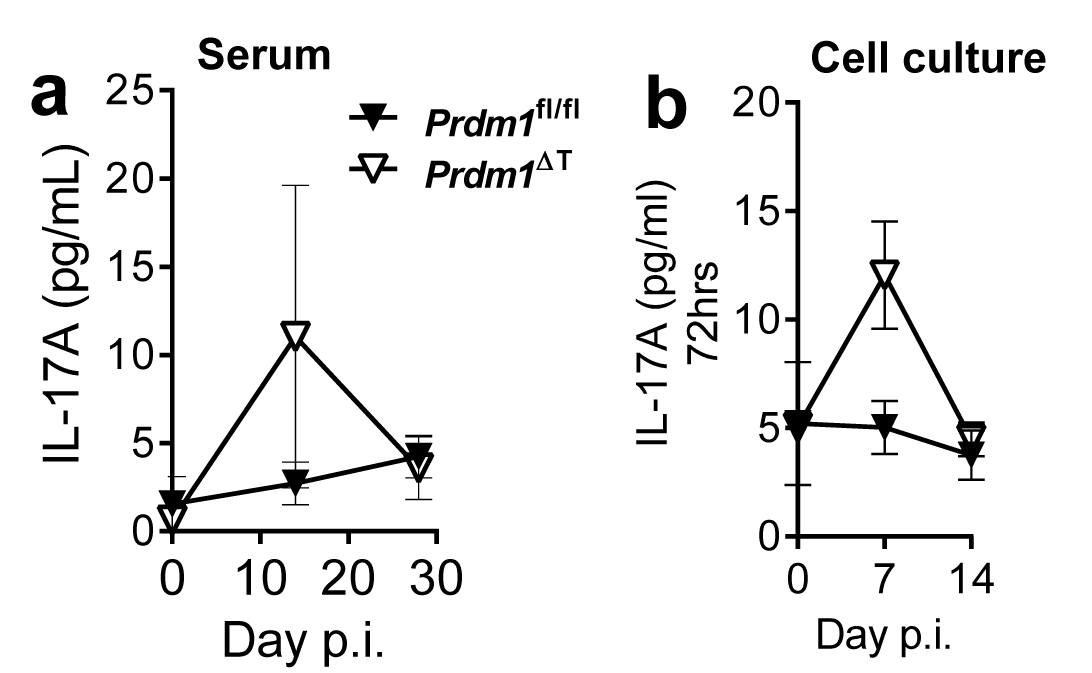

Supplement: S5 Fig — Prdm1 fl/fl and Prdm1 ΔT C57BL/6 mice were infected with L. donovani and serum IL-17A levels (A), as well as antigen-specific IL-17A production by splenocytes were measured after 72 hours of culture in the presence of parasite antigen (B) at times indicated. In both panels, closed shapes represent Prdm1 fl/fl mice, while open shapes indicate Prdm1 ΔT littermates. Representative of 2 similar experiments, mean ±SEM, n = 5–6 in each group in each experiment. (TIF) [file ppat.1005398.s005.tif]

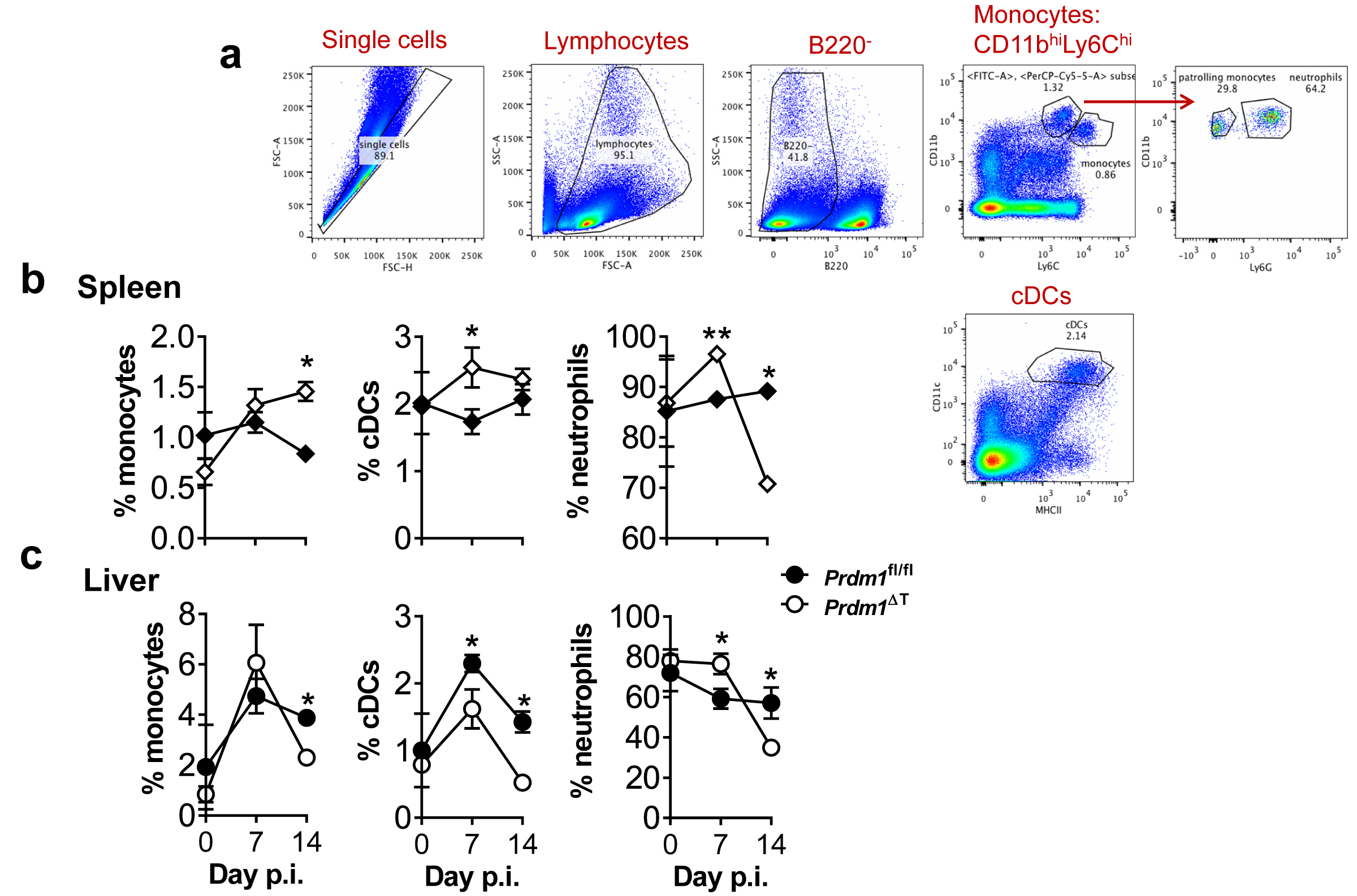

Supplement: S6 Fig — Prdm1 fl/fl and Prdm1 ΔT C57BL/6 mice were infected with L. donovani and monocytes, DC’s and neutrophils identified by the gating strategy shown in (A) and their frequency measured in the spleen (B) and liver (C) at time points indicated. In all panels, closed shapes represent Prdm1 fl/fl mice, while open shapes indicate Prdm1 ΔT littermates. Representative of 2 similar experiments, mean ±SEM, n = 5 in each group in each experiment, **p<0.01, *p<0.05, Mann-Whitney U test. (TIF) [file ppat.1005398.s006.tif]

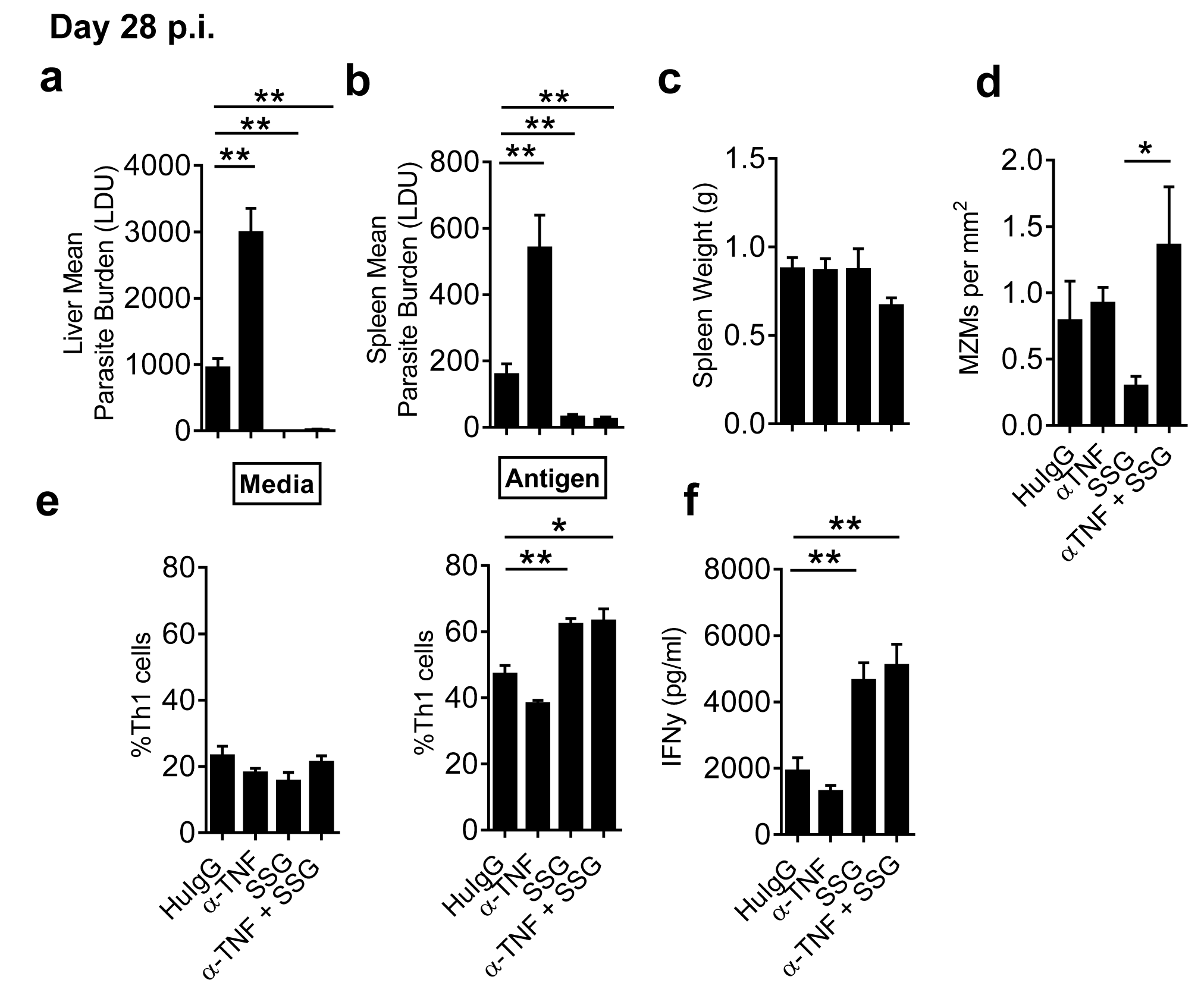

Supplement: S7 Fig — C57BL/6 mice were infected with L. donovani and received either TNF blockade (Enbrel) or control human IgG (INTRAGAM), with or without sodium stibogluconate (SSG), as indicated, from days 14–28 p.i.. Liver (A) and spleen (B) parasite burdens and spleen weights (C) were measured at day 28 p.i., as was the number of MZMs per mm2 of spleen tissue ((D); as described in Fig 4C). Th1 cell frequency in splenocytes cultured in media or with parasite antigen (E), as indicated, as well as IFNγ production from antigen-stimulated cells (F) were measured after 24 hours of culture. Representative of 2 independent experiments, mean ±SEM, n = 5, **p<0.01, *p<0.05, Mann-Whitney U test. (TIF) [file ppat.1005398.s007.tif]
